# A Review of Laboratory-Acquired Infections in the Asia-Pacific: Understanding Risk and the Need for Improved Biosafety for Veterinary and Zoonotic Diseases

**DOI:** 10.3390/tropicalmed3020036

**Published:** 2018-03-26

**Authors:** Jarunee Siengsanan-Lamont, Stuart D. Blacksell

**Affiliations:** 1 Animal Health and Laboratory Consultant, Perth, WA 6155, Australia; hjjar@yahoo.com; 2 Mahidol-Oxford Tropical Medicine Research Unit, Faculty of Tropical Medicine, Mahidol University, Bangkok 10400, Thailand; 3 Centre for Tropical Medicine & Global Health, Nuffield Department of Clinical Medicine, University of Oxford, Old Road Campus, Oxford OX3 7FZ, UK

**Keywords:** laboratory-acquired infection (LAI), Asia-Pacific, veterinary pathogens, zoonosis

## Abstract

A rapid review was performed to determine (1) the number and causes of reported laboratory-acquired infections (LAI) in the Asia-Pacific region; (2) their significance and threat to the community; (3) the primary risk factors associated with LAIs; (4) the consequences in the event of a LAI or pathogen escape; and (5) to make general recommendations regarding biosafety practices for diagnosis and research in the Asia-Pacific region. A search for LAI and zoonoses in the Asia-Pacific region using online search engines revealed a relatively low number of reports. Only 27 LAI reports were published between 1982 and 2016. The most common pathogens associated with LAIs were dengue virus, *Arthroderma* spp., *Brucella* spp., *Mycobacterium* spp., *Rickettsia* spp., and *Shigella* spp. Seventy-eight percent (21 out of 27 LAI reports) occurred in high-income countries (i.e., Australia, Japan, South Korea, Singapore, and Taiwan) where laboratories were likely to comply with international biosafety standards. Two upper-middle income countries (China (2), and Malaysia (2)) and one lower-middle income country (India (2)) reported LAI incidents. The majority of the reports (fifty-two percent (14/27)) of LAIs occurred in research laboratories. Five LAI reports were from clinical or diagnostic laboratories that are considered at the frontier for zoonotic disease detection. Governments and laboratories in the Asia-Pacific region should be encouraged to report LAI cases as it provides a useful tool to monitor unintended release of zoonotic pathogens and to further improve laboratory biosafety. Non-reporting of LAI events could pose a risk of disease transmission from infected laboratory staff to communities and the environment. The international community has an important and continuing role to play in supporting laboratories in the Asia-Pacific region to ensure that they maintain the safe working environment for the staff and their families, and the wider community.

## 1. Introduction

Working with pathogenic microorganisms requires good laboratory practices, risk assessments, and biosafety/biosecurity measures to ensure the safety of personnel, community, and the environment from accidental or deliberate infection. An occupationally-acquired infection of laboratory personnel is referred to as a laboratory-acquired infection (LAI) and these have been reported in scientific literature since 1897 [1]. Accidents or exposure events leading to LAIs may include inhalation of infectious aerosols, contact with mucous membranes by splash, touch, or spill, or infection via the percutaneous route (bites, cuts, accidental self-inoculation). However, in many LAI cases, the actual cause often remains unknown or uncertain [2,3,4].

There has been growing concern in the global scientific community and the general public regarding the potential for bioterrorism and accidental escape of pathogens from research laboratories. This concern has driven the debate regarding the restricting access to high-consequence pathogens and improving biosecurity measures, especially for those pathogens that have the potential to spread rapidly in the community [5,6,7]. Many experts consider the risk of pathogen escape to be low, especially in settings where there are well-developed regulatory environments combined with strong enforcement. However, to achieve this goal, diagnostic and research laboratories are required to implement and enforce strict biosecurity protocols and have well-trained personnel, especially those with biosafety level (BSL) 3 or higher containment level facilities [8].

The Asia-Pacific region is home to 4.5 billion people comprising almost 60% of the world population [9]. The region contains diverse economic conditions amongst member countries, ranging from high-income countries (HIC) (e.g., Australia, Japan) to upper-middle income countries (UMIC) (e.g., Republic of China, Malaysia) and lower-middle income countries (LMIC) countries (e.g., Cambodia, Laos PDR). The Asia-Pacific region is considered a disease hotspot, where laboratories that deal with human and veterinary pathogens have an important role in providing reliable services to support disease surveillance, diagnosis, prevention, treatment, research, and health promotion [10]. However, many laboratories of these laboratories are located in UMIC or LMIC, where biosafety infrastructure, regulatory practices, and enforcement of regulations may not exist or be as robust as in HICs.

The objectives of this rapid review were to determine (1) the number and causes of reported LAI in the Asia-Pacific region; (2) their significance and threat to the community; (3) the primary risk factors associated with LAI; (4) the consequences in the event of a LAI or pathogen escape; and (5) to make general recommendations regarding biosafety practices for diagnosis and research in the Asia-Pacific region.

## 2. Materials and Methods

### LAI Article Selection

This review aimed to summarize LAI reports from the Asia-Pacific region and to raise awareness of laboratory personnel of their occupational health and safety risks. The study used online search engines including PubMed, Google, Google Scholar, and the American Biological Safety Association (ABSA) LAI database (https://my.absa.org/LAI) to locate open sources of information on LAI reports or publications. The search was restricted to papers published in English up to 22 February 2018, combining the search terms ‘Asia’, ‘Pacific’ and ‘zoonosis’, ‘veterinary’ and ‘laboratory-acquired infection’ or ‘accidental infection’. The full texts of located articles were assessed to determine their suitability for inclusion. Reference lists of the located articles were also screened in order to identify additional studies. LAI reports from outside the Asia-Pacific region were excluded. Articles were screened by one author (JS-L) and discrepancies or queries were clarified with the second author (SDB).

## 3. Results

### 3.1. LAI Reports in the Asia-Pacific Region

A total of 27 LAI reports were published between 1982 and 2016 (Table 1). Fifty-six percent (15 reports) were reported in East Asia, 26% (7 reports) in Oceania, 11% (3 reports) in Southeast Asia, and 7% (2 reports) in South Asia. Seventy-eight percent (21 out of 27) of the reports were from developed countries, including Australia (26%; 7/27), Japan (15%; 4/27), South Korea (15%; 4/27), Taiwan (19%; 5/27) and Singapore (4%; 1/27). Nineteen percent of those LAIs (5 out of 27) were reported in China (7%; 2/27), India (7%; 2/27), and Malaysia (7%; 2/27).

Sixteen of the reports (59%) involved LAIs of bacterial origin, viruses were responsible for 33% (9/27), and fungi for 7% (2/27) of LAIs. The most commonly reported pathogens causing LAIs were dengue virus (3 reports), severe acute respiratory syndrome coronavirus (SARS-CoV) (3 reports), *Brucella* spp. (3 reports), *Arthroderma* spp. (2 reports), *Mycobacterium* spp. (2 reports), *Rickettsia* spp. (2 reports), and *Shigella* spp. (2 reports). Twenty-two LAI reports (81%) were zoonoses. Fifty-two percent (14/27) of LAIs occurred in research laboratories, seven (26%) from clinical/diagnostic laboratories and the remainder were unstated. The reported causes of the LAIs included human error (such as accidental self-inoculation, spills or cuts) although for 52% (14/27) the cause was unknown. Of the known LAI causes, six (22%) were suspected percutaneous infections, 11% (3/27) were splashes to the face, 7% (2/27) were possible aerosol exposures, (4%; 1/27) was a mosquito bite exposure, and 4% (1/27) was either an aerosol exposure or a mosquito bite.

### 3.2. Risk Assessment for Major Veterinary Pathogens in Asia-Pacific Region

Regions such as South and Southeast Asia are considered hotspots for emerging infectious diseases (EIDs). This is due to a combination of high-density human and animal populations, basic sanitation problems and inadequate health-related capacities [17]. The risk of infection is greatly increased by necropsy procedures, sample collection, clinical treatment, sample processing, and in vitro propagation. Specific control measures including personal protective equipment, and primary and secondary containment measures involving engineering controls are requires to mitigate risk of infection. Examples of emerging and zoonotic infectious diseases of high risk potential in Asia-Pacific region are presented in Table 2.

#### 3.2.1. High-Pathogenicity Avian Influenza (HPAI)

HPAI H5N1 viruses have recently caused disease outbreaks in poultry in Malaysia during 2017 [20] and Cambodia, Japan, Korea, and Taiwan during January 2018 [21]. Animal disease surveillance and monitoring activities have been routinely performed throughout Asia with a focus on HPAI virus and other transboundary diseases that affect international trade [22]. Processing samples originating from animal sources in a limited biosafety environment could pose a significant risk of LAI and could lead to unintentional release of the pathogen into environment by aerosol transmission when performing laboratory procedures [23].

In the case of potential pandemic pathogens, such as HPAI virus, existing wild type H5N1 viruses were reported to have a limited ability for human to human transmission [24] and the risk of the viruses crossing species boundaries to become a pandemic threat in humans was considered to be low [25,26]. However, evidence of a HPAI H5N1 strains, first detected in China in 2008, had undergone genetic re-assortment resulting in new NA subtypes (including N2, N3, N5, N6, and N8) and aggressively spread to birds worldwide (e.g., Asia (mainly H5N6), Europe (H5N8), Middle East (H5N8), Russia/Mongolia (H5N8) and North America (H5N8, H5N1 and H5N2)) [27]. These H5Nx viruses are believed to become more transmissible and more stable in the environment and in wild birds than other influenza A viruses [27]. Similar to the H5Nx, H7N9 low-pathogenicity avian influenza (LPAI) first caused human infection in 2013 in China [28]. Since then, the viruses recurred annually and caused 1589 human cases with 616 deaths [29]. It is unclear how mutation and re-assortment occur in nature [27,30]. Influenza viruses are well known for their fast rate of genetic re-assortment especially when co-infection has occurred [31]. There is a possibility that these viruses could undergo genetic assortment and infect humans leading to a potential pandemic [30]. The viruses that have been circulating in the region persist in the environment and infect a range of hosts posing a risk of generating a potential pandemic strain [32].

#### 3.2.2. *Brucella*

Brucellosis is one of the main causes of LAIs [33] and between 1979 and 2015, brucellosis was reported as causing 378 LAIs [34]. A study by Traxler et al. [35] reported that of 167 potential *Brucella*-exposed workers, 71 developed LAI brucellosis. Aerosolization was associated with 88% of the cases and 11% were exposed by accidents [35]. *Brucella melitensis* was the causative agent in 80% of LAIs [35]. In the Asia-Pacific region, brucellosis is endemic and the predicted prevalence of the disease in livestock ranges between 3% for South East Asia and 16% for South Asia [36], posing a high risk of exposure to veterinary laboratory workers.

#### 3.2.3. Rabies

Rabies has been estimated to infect as many as 31,000 humans annually in Asia [37]. In India alone, there were approximately 20,000 cases annually, which was one-third of the rabies cases reported worldwide [38]. Even though laboratory-acquired rabies is rare, the mortality rate of the infected cases is 100% in the case of an untreated exposure [39]. High disease prevalence combined with increased exposure risk through diagnostic activities with poor biosafety practices could increase the potential of LAIs to laboratory personnel. Pre-exposure vaccination, good laboratory practices, effective biosafety measures, and post-exposure prophylaxis (PEP) treatment are key to protecting those who work with the rabies virus.

#### 3.2.4. Other Zoonotic Pathogens and Major Transboundary Livestock Diseases

Other zoonotic pathogens of viral origin that have recently emerged or are endemic in South Asia, Southeast Asia and Oceania include Nipah/Hendra [40,41] and Japanese encephalitis viruses [42]. Bacterial diseases of zoonotic concern include *E. coli*, *Pasteurella multocida*, *Salmonella* serovar Enteritidis, *Bacillus anthracis*, *Streptococcus suis*, and *Coxiella burnetii*. Precautions and strict biosafety measures should be applied when working with exotic animal or wildlife samples especially bats, due to the potential carriage of unknown pathogens including EIDs. Major transboundary livestock diseases including classical swine fever virus (CSFV), foot and mouth disease virus (FMDV), porcine respiratory and reproductive syndrome (PRRS) virus are also endemic in South and Southeast Asia requiring laboratory diagnosis, propagation, and certain conditions increasing the risk of accidental exposures and release.

## 4. Discussion

This review summarized LAI reports from the Asia-Pacific region and has examined some of the potential risks associated with laboratory investigations with zoonotic pathogens. Clinical diagnoses and routine disease surveillance and monitoring activities comprise a major workload for health-related laboratories at both public and animal health interfaces in the region. Working with infectious materials on a daily basis poses a risk to laboratory staff health as well as those involved in the collection and transportation of samples. Accidental infection of laboratory staff can occur even in laboratories where strict biosafety measures are employed and enforced as demonstrated by those that occurred in laboratories located in HIC [43,44,45]. Reporting of LAIs can be an indication of a biosafety or biosecurity breach that may be caused by technical failures or human errors. Thorough investigations to determine the root cause of LAIs or unintended releases have the potential to improve laboratory biosafety by providing an evidence base to determine risks of such occurrences. 

A series of landmark studies of LAI occurrence and cause were performed in the USA by Sulkin and Pike between 1935–1978 [46,47] with a total of 4079 LAI reports that that concluded that the majority of LAIs were caused by bacterial pathogens with lower numbers of viral and rickettsial infections. In another study, Pike claimed that only 64% of LAI reports were published (2465 out of 3921 cases; data collected between 1935 and 1974) [2]; however, these were based mostly on LAIs from research and animal laboratories and did not represent cases in clinical laboratories [43]. In 2017, Byers and Harding [34] stated that, based on their review study, 43% (out of 2308) of LAIs occurred in clinical laboratories and 39% in research laboratories.

Most LAI reports in this study were from developed HIC, where biosafety measures are more likely to be compliant with international standards, and which report LAIs as per national regulatory requirements. Furthermore, there may be an incentive to report LAIs in HICs due to the requirement for biosafety competency of staff coupled with an awareness of potential LAI hazards, and the need to report accidents so that appropriate post-exposure treatment could be provided in a timely manner. In comparison to other parts of the world, the number of LAI reports in the Asia-Pacific was relatively low. This may be due to lower numbers of laboratories in Asia, especially those that would normally be handling high-consequence pathogens, such as high-containment laboratories (i.e., BSL3 and BSL4). Furthermore, the lower number of LAIs may also be due to different reporting requirements for research, diagnostic, or clinical laboratories, and whether they are government or privately-funded, and the fear of stigma [4,43]. A limitation of this review is that searches were only performed using searchable sources and that the authors did not have access to information such as locally published unofficial reports, or those in languages other than English, which may also have contributed to the low number of LAI reports. Nevertheless, it is likely that there is significant under-reporting of LAIs in the UMIC and LMIC of the Asia-Pacific, given the significant disease risk profile. Under-reporting and lack of recognition of such LAIs could pose risk not only to staff but also the community and the environment.

Working with ministries of health, ministries of agriculture, and other governing bodies within the UMIC and LMIC of the Asia-Pacific region to set up mandatory LAI reporting, as well as accidental release or escape, would provide evidence of capacity gaps and where to focus biosafety resources; however, these data have limitations. The downside of using LAIs reports to determine capacity gaps is that they are an insensitive method of detecting laboratory exposure, because they are based on acute symptomatic infection, while data on asymptomatic infection and host immune response are rarely measured to determine seroconversion status [33]. A survey by Willemarck et al. [48] commented that the majority of LAI reports and publications only identified the most obvious risk group 3 organism LAIs and risk group 2 organisms, which cause milder or asymptomatic infection, were unlikely to be detected as the resulting LAIs would be unknown or unnoticed [48]. Lack of awareness could result in low or no LAI reporting in some laboratories. In the US, a mathematical model determined that the probability of LAI incidents was between 0.1 and 0.5% [49], which was similar to the probability of an annual pathogen escape at 0.3% [50], although it is not clear how these data would relate to the Asia-Pacific setting. Therefore, it is important that all laboratory staff are educated in accident and incident reporting, as well as following up cases of exposure to pathogens with post-exposure prophylaxis. Additionally, a registry documenting the near-miss incidents would help to improve laboratory safety.

Results of Asia-Pacific LAIs reports presented here share some characteristics with those previously published. Elsewhere, LAI reports have generally decreased due to increasingly effective vaccines along with better laboratory safeguards; however, the risk of laboratory exposure and escape still remains when working with live agents [51]. A literature review of LAI reports in the USA during 2000–2009 revealed that there was a total of 34 cases with four deaths, due to bacteria (22) and viruses (11) and parasites (1) [4]. According to the *Monitoring Select Agent Theft*, *Lost and Release* reports in the USA (2004–2010), 11 LAIs were reported with no fatalities or evidence of secondary infection to others [52]. However, it should be noted that these reports were confined to Select Agents and not all possible pathogens. There were six cases of brucellosis, four cases of *Francisella tularensis*, and one case of *Coccidioides immitis/posadasii* [52]. A survey in Belgium indicated that between 2007 and 2012 there were 94 LAI cases; 23% caused by *Salmonella* spp. and 16% by *Mycobacterium* spp. In 2000, Sewell [48] reported that the most common organism causing LAIs included bacteria (*Shigella* spp., *Salmonella* spp., *E. coli*, *Francisella tularensis*, *Brucella* spp., *Mycobacterium tuberculosis*), viruses (hepatitis C virus, human immunodeficiency virus), and a dimorphic fungus. To date, even though pathogens listed above by Sewell remain the primary cause of LAIs, other organisms including *Neisseria meningitidis* and vaccinia virus, as well as newly-emerged pathogens with a potential pandemic risk (e.g., SARS, influenza viruses, West Nile virus, and Ebola virus) should also be considered as significant potential pathogens for LAIs [48].

To reduce the likelihood of LAI occurrences, it is important that each laboratory plan and implement their own pathogen-specific, preventative strategies to improve biosafety and biosecurity. This includes development and application of protocols specific for occupational health and safety incorporating accident reporting and ‘close call’ incidents, and pre/post-exposure serological surveys. When working with pathogens, a risk-based approach should be applied for all biosafety programs focusing on pathogen-based factors. The factors to be considered are routes of infection, infectious dose, quantity and concentration of the agent to determine the most appropriate risk mitigation strategies, such as administrative and engineering controls, and personal protective equipment [53]. Furthermore, annual health checks and vaccinations, post-exposure prophylaxis—including reporting and monitoring for post-vaccination adverse events—and symptom monitoring, are recommended [54,55].

In conclusion, clinical and diagnostic laboratories are on the front line for detecting outbreaks of EIDs and zoonotic diseases. Laboratories require strong biosafety measures to protect staff health and prevent environmental contamination with pathogens. The fundamentals of a biosafety program include staff education and awareness to ensure good understanding and implementation of biosafety measures, including risk assessment and control measures [56]. The international community has an important and continuing role to play in supporting laboratories in UMIC and LMIC to ensure that they maintain a safe working environment for the staff, their families, and the wider community.

## Figures and Tables

**Table 1:** Summary of the LAI reports in the Asia-Pacific region [11].

Year	Location	Agents Involved	Possible Cause	Affected Personnel	Type of Laboratory
2016	Taiwan	*Ralstonia pickettii*	Unknown	-	-
2014	South Korea	Dengue	Self-inoculation	Laboratory staff	Research/BSL2
2011	Australia	Dengue	Mosquito-bite or aerosol	Scientist	Research/BSL2
2010	India	Buffalopox virus (BPXV) (Z)	Broken ampoule	Researcher	-
2009	Malaysia	*Brucella melitensis*	Unknown	Laboratory staff	Clinical
2006	Taiwan	*Shigella* spp. (Z)	Unknown	Graduate student	Research
2006	PR China	Seoul virus and hantavirus (Z)	Possible aerosol	8 postgraduate students	Research
2004	Taiwan	Dengue type 1	Mosquito bite	Graduate student	Research
2004	Taiwan ′	SARS-CoV (Z)	Cleaning spilled waste	Researcher	Research
2004	PR China ″	SARS-CoV (Z)	Unknown	8 human cases, 1 died	Research
2003	Singapore	SARS-CoV (Z)	Unknown	Graduate student	Research/BSL3
2002	Japan	*Arthroderma benhamiae* (Z)	Unknown	Scientist	Research
2002	Australia	*S. aureus*, MRSA, EMRSA (Z)	Wound contamination	Laboratory staff	Clinical
2001	Japan	*Arthroderma benhamiae* (Z)	Unknown	Researcher	Research
2000	South Korea	*Orientia tsutsugamushi* (Z)	Possible aerosol	Worker	-
1999	Taiwan	*Vibrio parahaemolyticus* (Z)	Handled infected abalones	Laboratory staff	-
1998	Japan	*Helicobacter pylori* (Z)	Unknown	Bacteriologist	-
1996–2000	Australia	*Brucella suis* (Z)	Unknown	Various	Clinical
1996	Malaysia *	*Salmonella typhi*	Unknown	Laboratory staff	-
1992	Australia	*Pseudomonas pseudomallei* (Z)	Unknown	3 laboratory staff	Diagnostic
1990	South Korea	*Rickettsia typhi* (Z)	Unknown	Laboratory staff	Clinical
1990	India	*Mycobacterium leprae* (Z)	Unknown	Worker	Clinical
1989	South Korea	*Rickettsia typhi* (Z)	Splash to face	Laboratory staff	Research
1987	Australia^	Newcastle disease virus (Z)	Splash to face	Laboratory staff	Research/BSL3
1986	Australia^	*Brucella melitensis* (Z)	Accidental self-inoculation	Researcher	Research
1985	Japan	*Mycobacterium tuberculosis* (Z)	Unknown	Pathologist	Research
1982	Australia	*Shigella flexneri* (Z)	Splash to face	Laboratory staff	Clinical

* [12]; ^ [13]; ′ [14]; ″ [15]; Z–Zoonoses or potential zoonoses, ~ unidentified year: a review study performed on data collected between 1996 and 2009 [16].

**Table 2:** Emerging and zoonotic infectious diseases of high risk potential in Asia-Pacific region [18].

Infectious Agent	Risk Group *
*Virus*	
Avian influenza	2/3
Chikungunya	3
Crimean-Congo hemorrhagic fever	4
Dengue	2
Ebola virus disease	4
Hantavirus	3
Japanese encephalitis	2
Nipah virus	4
Novel human coronavirus (SARS)	3
Rabies	3
Viral hepatitis	2
*Bacteria*	
Anthrax	3
Brucellosis	3
Leptospirosis	2
Listeriosis	2
Melioidosis	3
Plague	3
Salmonellosis	2
Scrub typhus	3
Tularaemia	2/3
*Parasite*	
Taeniasis/cysticercosis	2
Toxoplasmosis	2
Trichinellosis	2

* Based on the risk group database of ABSA.org [19].
